# Expression and Clinical Significance of Antiapoptotic Gene (Survivin) in NB4 and Acute Promyelocytic Leukemia Cells

**DOI:** 10.1100/2012/937087

**Published:** 2012-04-01

**Authors:** Jun Xue, Xiao-Jing Xie, Mao-Fang Lin

**Affiliations:** ^1^Department of Hematology, Nanjing First Hospital Affiliated to Nanjing Medical University, NanJing 210006, China; ^2^Department of Hematology, First Affiliated Hospital, Zhejiang University College of Medicine, Hangzhou 310009, China

## Abstract

To study survivin gene expression in APL cells and to explore its correlation with clinical manifestations. PML/RAR*α* and survivin mRNA expression were analysed using RT-PCR. By treatment of ATRA, the survivin mRNA expression in NB4 cells gradually decreased with time and was almost undetectable in the 72th hour. Survivin was expressed in 67% of the 36 APL cases (de novo and relapse patients) with PML/RAR*α* fusion gene expression. However, in 22 cases of remission stage patients without PML/RAR*α* fusion gene expression, survivin was expressed in 36%. The survivin mRNA expression positive rate in de novo and relapse groups, and PML/RAR*α* fusion gene L-type positive groups, was obviously higher than those in remission period groups and was significantly lower than those in acute leukemia groups. In 36 cases of de novo and relapse APL patients, all cases could obtain complete remission, irrespective of the survivin expression. APL patients expressed with survivin mRNA had DIC and serious infection (one patient died). The clinical symptom included slight skin or mucosa bleeding, fever, and asthenic for patients without the survivin mRNA expression. Later, two cases of APL patients with the survivin mRNA expression were treated by ATRA, induction differentiation sign in their peripheral blood and bone marrow figure was not obvious. It was concluded that the survive gene expression was lower in APL than those in any other types of leukemia, thus closely associated with clinical manifestation.

## 1. Introduction

It has been shown that PML-RAR chimeric protein, as a result of the specific chromosome translocation t(15; 17), plays a central role in acute promyelocytic leukemia (APL) pathogenesis. Clinically, APL is sensitive to antileukemic chemotherapy, to the differentiation therapy with all-trans retinoid acid (ATRA), and to arsenic compounds including arsenic trioxide (As_2_O_3_) and arsenic disulfide.

Several multicenter clinical trials combining ATRA and chemotherapy have further improved the survival of APL patients. Arsenic compounds have allowed not only a high complete remission (CR) rate, but also relatively long-term survival in a significant proportion of patients.

Survivin, a member of the inhibitors-of-apoptosis (IAP) family of proteins, is present during fetal development but is undetectable in terminally differentiated adult tissues. However, survivin is prominently expressed in transformed cell lines, in most common human cancers, and in approximately 50% of high-grade non-Hodgkin lymphomas. Survivin suppresses apoptosis induced by Fas, Bax, caspases, and anticancer drugs. Conversely, the downregulation of survivin by antisense oligonucleotides induces apoptosis in vitro. Although survivin protein lacks the ability to directly inhibit caspase-3, it binds quantitatively to a new IAP-inhibiting protein, Smac/Diablo, raising the possibility that it might suppress caspases indirectly by freeing other IAP family members from the constraints of this protein. Taken together, these studies support the notion that survivin exerts an antiapoptotic effect.

Our prior research report showed that survivin gene expression levels in acute leukemia cells were significantly higher than those in normal bone marrow mononuclear cells (82% versus 33.3%, *P* < 0.05). Among 5 cases of APL, 4 cases did not express survivin. In 4 cases of APL, which the survivin mRNA expression were negative, after being treated by ATRA, 3 cases of APL achieved bone marrow remission (BMR), the APL patient of survivin positive expression died of DIC [1]. Accordingly, is there obvious difference between APL and other leukaemia in survivin genetic transcription level? In the present study, survivin transcription level in APLs had been investigated by means of a reverse transcriptase polymerase chain reaction (RT-PCR) analysis. Furthermore, the prognostic values of survivin expression in these patients were also assessed and evaluated.

## 2. Methods

### 2.1. NB4 Cell Line Culture

Human NB4 cell lines of APL was cultured in RPMI1640 (Invitrogen, USA) medium containing 10% newborn calf serum in a humidified atmosphere of 95% air/5% CO_2_ at 37°C. To minimize spontaneous nucleosomal DNA fragmentation and apoptosis, cells were maintained at densities <5 × 10^5^ cells/mL by adjusting cell concentrations daily with adding fresh culture medium and corresponding concentrations of compounds while necessary. NB4 cells (1 × 10^5^ cells/mL) were treated with ATRA (1 *μ*mol/L) for 24, 48, 72 hours and then harvested. The survivin mRNA expression were analysed using reverse transcriptase polymerase chain reaction (RT-PCR) technique.

### 2.2. Patient Characteristics and Primary Samples

There were 36 APL patients hospitalized or served with out-patient clinic (34 newly diagnosed and 2 relapsed). The patients aged from 9 to 72 years (average, 40.5 years); there were 17 men and 19 were women. The remission period of APL patients involved 22 cases. The patients aged from 3 to 57 years (average, 30 years) among 10 male and 12 female, the remission duration lasted from 1 to 72 months (average, 37 months). Diagnosis was based on MICM typing standard (among which 20 cases were M_3a_ and 16 M_3b_). Patients of de novo APL were first treated with ATRA. When the peripheral white blood cells ascend to 10.0 × 10^9^/L, HA (homoharringtonine and cytarabine) chemotherapy regimen were used. The APL patients of relapse and ATRA drug resistance were applied with As_2_O_3_. The bone marrow remission rate (BMR) was acted as therapeutic effect index. The acute leukemia (AL) groups were 23 cases (M_1, _3, M_2, _6, M_4-5, _2, ALL-L_1, _4, ALL-L_2, _8). Patient aged from 20 to 64 years (average, 42 years); there were 16 men and 7 women. 

Bone marrows were obtained from patients with APL and normal donors after informed consents were obtained according to institutional guidelines. Mononuclear cells were purified by Ficoll-Hypaque (Sigma Chemical, St Louis, MO) density-gradient centrifugation.

### 2.3. RNA Extraction and PCR Amplification of PML-RAR-*α* [2] and Survivin RNAs

The concrete procedure was shown in [2] 2 × 10^6^ NB4 cells were twice washed with PBS solution, and RNA was isolated with Trizol solution (Gibco, USA), according to the manufacturer's instructions, eluted with 50 *μ*L of diethylpyrocarbonate water containing 50 units of RNase inhibitor (Boehringer Mannheim, Indianapolis, IN). RNA concentration was determined by spectrophotometry and adjusted to a concentration of OD_260_/OD_280_ ≥ 1.7.

Two microgram total RNA cases were reverse transcribed with survivin. RNA from NB4 cells and bone marrow mononuclear cells of APL patients were purified by using the Ultraespec RNA kit (Biotecx Laboratories, Houston, TX), according to the manufacturer's instructions, and eluted with 50 *μ*L of diethylpyrocarbonate water containing 50 units of RNase inhibitor (Boehringer Mannheim, Indianapolis, IN). RNA was reverse transcribed by Moloney murine leukemia virus reverse transcriptase (Promega company) and 0.5 *μ*g oligo(T)18. Incubation was performed at 70°C for 5 min, 42°C for 60 min, and 70°C for 15 min. CDNA was then amplified by PCR cDNA 2 *μ*L, 20 pmol/L forward and reverse primer 1 *μ*L each, 10 mM dNTP 0.5 *μ*L, 10 × PCR buffer 2.5 *μ*L, 25 mM MgCl 21.5 *μ*L, TaqDNA polymerase 2 U, total volume 25 *μ*L). The sequence of the sense primer of survivin was 5′-ctttctcaaggaccaccgcatc-3′ and the antisense primer was 5′-caatccatggcagccagctgc-3′ as of survivin. Cycling conditions were as follows: initial denaturation at 94°C for 2 min, followed by 39 cycles at 94°C for 45 seconds, 61°C for 1 min, 72° for 1 min, and finally 72°C for 5 minutes. Identical PCR conditions were used to amplify the beta-actin cDNA that was used as an internal control; the sense and antisense primer sequences used for beta-actin were 5′-cgctgcgctggtcgtcgaca-3′ and 5′- gtcacgcacgatttcccgct-3′. PCR amplifications were performed in duplicate using independent cDNA preparations. Tubes containing all ingredients except for the cDNA templates were incubated in all runs and served as negative controls. Amplified cDNAs were separated on 1.5% agarose gels, and the bands were visualized by ethidium bromide and photographed with a Polaroid camera (Polaroid, Cambridge, MA).

### 2.4. Statistical Analysis

The SPSS 10.0 software for Windows was used for analysis. The statistical significance of intergroup differences was evaluated by Fisher's exact test. Values of *P* < 0.05 were considered statistical significance.

## 3. Results

### 3.1. Expression of Survivin mRNA in NB4 Cells Treated with ATRA

**Figure 1:**
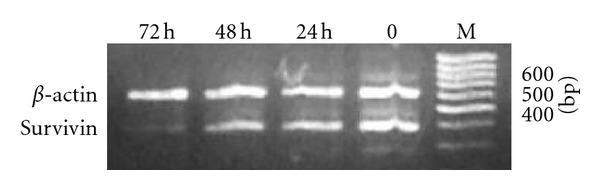
Survivin mRNA expression of NB4 cells treated by ATRA at different time.

As shown in Figure 1, the survivin mRNA expression was detected in NB4 cells. By treatment of 1 *μ*mol/L ATRA, survivin mRNA expression in NB4 cells gradually decreased with time and almost undetectable in the 72th hour point.

### 3.2. Expression of Survivin mRNA in the de Novo, Relapse Groups and Remission Period Groups of APL Patients

The results were shown in Table 1. The positive and negative rates of survivin mRNA expression were 67% and 33%, respectively, in all 36 cases of de novo and relapse APL patients whose PML/RAR_*α*_ fusion gene expression was positive. There were also 22 cases of remission patients whose PML/RAR_*α*_ fusion gene expression was negative, and the positive and negative rates of survivin mRNA expression were 36% and 64%, respectively. The survivin mRNA expression positive rate in the de novo and relapse groups, and PML/RAR_*α*_ fusion gene L-type positive groups was obviously higher than those in remission period groups (*P* < 0.05) and was obviously lower than those in acute leukemia grougs (*P* < 0.05, <0.001).

### 3.3. The Relation between Survivin mRNA Expression and Clinical Manifestation in APL Patients

Regardless the survivin mRNA expression was positive or negative in 36 cases of de novo and relapse APL patients, all of 36 cases could obtain complete remission. The APL patients with positive survivin mRNA expression had combination of DIC and serious infection (one patient died). The clinical symptom had slight skin or mucosa bleeding, fever, and asthenic for patients without the survivin mRNA expression. Afterwards 2 APL patients with the survivin mRNA expressed were treated by ATRA, induction differentiation sign in their peripheral blood and bone marrow figure was not obvious. It was concluded that the survivin gene positive expression rate was lower in acute promyelocytic leukemia than those in any other types of leukemia and was related to clinical manifestation.

## 4. Discussion

First of all, the survivin mRNA expression of bone marrow mononuclear cells in acute leukemia (except APL) was examined. The result showed that the survivin mRNA was expressed in out of 23 cases (91%), which was consistent with Carter et al'.s [3] report showing the survivin expressed in 88.9% of acute myelocytic leukemia (AML).

Our earlier research has demonstrated that four of five patients with APL who did not express survivin in the bone marrow mononuclear cells had milder clinical symptoms. The patient died of DIC eventually [1]. Is it true that his cell biological behaviour of APL is significantly unique? 

It is known that APL represents a unique model in terms of the biological and clinical features. The complete remission rate reachs up to 90% after differentiated therapies with all-trans retinoid acid (ATRA). Arsenic compounds including arsenic trioxide (As_2_O_3_) and arsenic disulfide had allowed not only a high complete remission (CR) rate, but also relatively long-term survival. Are these phenomenons associated with antiapoptotic gene surviving? Up to now, few reports are available about survivin gene expression in APL. The survivin mRNA expression of bone marrow mononuclear cell was analyzed in 36 cases of de novo and relapse APLs. As bone marrow samples from other types' acute leukemia were also analyzed, the result indicated that survivin mRNA expression rate of de novo and relapse APLs were obviously lower (67% versus 91%, *P* = 0.03), more lower in remission patients (67% versus 36%, *P* = 0.03). 

Many research results have demonstrated that the survivin gene expression in tumour tissue is correlated to clinic prognosis. A potential distribution and prognostic significance of survivin in patients with de novo acute myeloid leukaemia (AML) was investigated by Adida et al. [4]. By immunofluoresence of bone-marrow specimens and peripheral blood mononuclear cells, survivin was detected in 75 out of 125 interpretable AML cases (60%). There was no significant difference in complete remission rate or overall survival between survivin-positive and survivin-negative AML patients. However, survivin expression became an independent prognostic factor for survival. These data suggest that survivin expression may be considered as a new unfavourable prognostic factor of de novo AML and suggest a role for apoptosis inhibition in influencing disease outcome. Of the 222 patients with diffuse large B-cell lymphomas studied [5], 134 (60%) revealed survivin expression in virtually all tumor cells by immunohistochemistry. The overall 5-year survival rate was significantly lower in patients with survivin expression than in those without. Multivariate analysis incorporating prognostic factors from the International Prognostic Index (IPI) identified survivin expression as an independent predictive parameter on survival in addition to LDH, stage, and ECOG scale.

A second analysis incorporating IPI as a unique parameter demonstrated that survivin expression remained as a prognostic factor for survival independently of IPI. Survivin expression may be considered a new unfavourable prognostic factor of diffuse large B-cell lymphoma. 36 cases of initial relapse APL and 22 cases of remission were followed up for more than three years. Because of deficient time, the correlation between survivin mRNA expression and clinical prognosis was not observed.

Our study indicated that the survivin over-expression was detected in NB4 cells. By treatment of ATRA, survivin mRNA expression in NB4 cells gradually deceased with time and almost could not be detected in the 72th hour. The other research had shown that cytokine stimulation increased survivin expression in leukemic cell lines and in primary AML samples [3]. In cultured primary samples, single-cytokine stimulation substantially increased survivin expression in comparison with control cells, and the combination of G-CSF, GM-CSF, and SCF increased survivin levels even further. Conversely, all-trans retinoic acid significantly decreased survivin protein levels in HL-60, OCI-AML3, and NB-4 cells within 96 hours paralleled to the induction of myelomonocytic differentiation. These results demonstrate that hematopoietic cytokines exert their antiapoptotic and mitogenic effects and that survivin obviously correlates with cell differentiation. Accordingly, it was concluded that if tumour cells were induced and differentiated to mature, the survivin mRNA expression would have been downregulated. The differentiation level of APL cells is higher than other types' leukemic cells. Accordingly, the survivin mRNA expression of APL cells is lower. The researcher has proved that ATRA treatment prior to chemotherapy may improve the CR rate in patients with de novo AML, which seems to be related to its beneficial effect on multidrug resistance [6].

## Figures and Tables

**Table 1:** The results of survivin mRNA expression in de novo, relapse, and remission APL.

Group	*N*	survivin-mRNA expression	
		positive	*P* value
(1) de novo and relapse*	36	24 (67%)	
L type (+)^#^	28	19 (68%)	
S type (+)	8	5 (63%)	
(2) remission^&^	22	8 (36%)	
(3) acute leukemia	23	21 (91%)	

Compared by remission *x ^2^ = 5.07, *P* = 0.024; ^#^ *x* ^2^ = 4.92, *P* = 0.027; compared by acute leukemia *x ^2^ = 4.71, *P* = 0.03; ^&^ *x* ^2^ = 14.81, *P* = 0.00.
